# Understanding Adolescents' Perceptions of Diarrhea: A Formative Research Study of a Visual Scale to Measure Self-Reported Diarrhea in Low-Resource Settings

**DOI:** 10.3389/fpubh.2021.561367

**Published:** 2021-05-25

**Authors:** Anise Gold-Watts, Geir Aamodt, Subramanian Gandhimathi, Rajamani Sudha, Sheri Bastien

**Affiliations:** ^1^Department of Public Health, Faculty of Landscape and Society, Norwegian University of Life Sciences, Ås, Norway; ^2^Department of Community Health, Sri Narayani College and School of Nursing, Vellore, India; ^3^Department of Obstetrics and Gynecology Nursing, Sri Narayani College and School of Nursing, Vellore, India; ^4^Department of Community Health Sciences, Cumming School of Medicine, University of Calgary, Calgary, AB, Canada

**Keywords:** adolescent, diarrheal illness, Bristol Stool Form Scale, illness representations, water sanitation and hygiene

## Abstract

**Introduction:** Although water, sanitation, and hygiene interventions are effective in reducing diarrhea, there are methodological issues regarding the research tools used to evaluate their health impact. Moreover, there is limited research on individuals' subjective interpretations of diarrheal illness which may introduce further limitations in relying on self-reported data. Therefore, we conducted a study that aims to understand adolescents' perceptions of diarrheal illness in rural Tamil Nadu, India. Next, we wish to explore the acceptability of the Bristol Stool Form Scale to assess self-reported diarrhea in water, sanitation, and hygiene interventions involving adolescent participants in low-resource settings.

**Materials and Methods:** The study was conducted as part of the formative research phase in the cultural adaptation of Project SHINE, a school-based educational water, sanitation, and hygiene intervention in Thirumalaikodi, Tamil Nadu, India. A convergent parallel mixed-methods study design with a purposive sampling strategy was used. Qualitative data included 10 in-depth interviews with student participants aged 13–14. Quantitative data were collected through interviewer-administered face-to-face surveys (*n* = 14) and one-week stool diaries (*n* = 14). Each data set was analyzed separately and compiled during the interpretation of the findings.

**Results:** Across all data sets, diarrhea was reported to be perceived as unhealthy and an irregular occurrence among participants. Participants also reported diarrheal-taboos, local methods to cure or control diarrhea, and discussed how diarrheal illness can lead to absenteeism or withdrawal from school and social activities. Moreover, participants were able to understand and answer questions about their stool using the Bristol Stool Form Scale, suggesting that is an acceptable tool.

**Discussion:** Visual tools demonstrate promise in improving self-reported diarrheal illness among adolescents in low-resource settings in India. However, until we address diarrhea-related taboos it will be difficult to address methodological issues in the assessment and reporting of diarrheal illness among adolescents.

## Introduction

Diarrheal disease is often caused by bacterial, viral, or parasitic organisms, due to unsafe drinking water, inadequate sanitation, or poor hygiene (1–4). Globally, three out of ten people do not use safely managed drinking water and an estimated six out of ten lack access to sanitation services (5). The population most affected by diarrheal disease are children under five in low- and middle-income countries (6, 7), however children older than 5 years, adolescents, and adults also experience more than 2.8 billion episodes of diarrhea per year (8), demonstrating how diarrheal disease affects the mortality and morbidity of individuals in all age groups (9, 10). Furthermore, even though few studies have been conducted on the health and social consequences of diarrhea among adolescents, researchers connect diarrheal disease among adolescents to school absences and hospitalization (8, 11).

In countries such as India, diarrheal disease claims the lives of approximately 300,000 children under 5 each year (12). Furthermore, India has one of the largest populations globally, which lack access to clean drinking water, and sanitation coverage (13). As of 2015, 66% of the population in India still lacks access to basic sanitation and 22% lack access to basic drinking water (13), illustrating how water, sanitation, and hygiene (WASH) continues to be a major public health challenge in this context.

Systematic reviews have demonstrated that WASH interventions are effective in reducing diarrheal disease (14, 15); however, there are still lingering methodological issues pertaining to the research tools used to evaluate health impact in low-resource settings. In WASH interventions, data collection tools to investigate diarrheal disease often include objective measurements such as stool collection (assessing enteric pathogens), observation (caregiver reports of frequency/consistency), and self-report. However, stool collection is costly, requires expertise, facilities, storage, and has several other logistical limitations (16), while caregiver reporting can be unreliable (17). Furthermore, evidence of the impact of WASH interventions on child health is mixed, since, many studies rely on self-reported symptoms, which can be susceptible to biases (recall, social desirability, and measurement error) (16, 18–20). Despite evidence suggesting that self-reported outcome measures are unreliable, studies investigating the impact of WASH interventions continue to rely on them.

There is also a lack of conceptual clarity concerning diarrhea (17, 19–23) and several definitions of diarrhea exist to assist with the diagnosis. Researchers and practitioners tend to use the World Health Organization's (WHO) definition, which is based on a study of children in Bangladesh (23). The WHO defines diarrhea as three or more loose or watery stools in 24 h (24). However, individuals may have different subjective interpretations surrounding the definition of diarrhea, which introduces methodological challenges in self-reporting (16, 22, 25). Subjective interpretations of diarrhea may be informed by their unique cultural context; therefore, it is difficult to determine a single unanimous understanding of diarrhea between researchers, practitioners, and study participants. Finally, such discrepancies in the understanding of diarrhea may also have profound consequences on how the impact of an intervention is understood when diarrhea is a primary outcome.

In a recent review of 55 WASH interventions with the intended purpose of reducing diarrheal disease, 36 studies applied the WHO definition (26). Despite the widespread use of the WHO definition, there is still limited research exploring cultural and contextually influenced diarrheal illness representations (19). Researchers must acknowledge these limitations when using self-report instruments in WASH research, given that diverse representations and perceptions of diarrheal illness held by the population of interest may introduce measurement errors. This demonstrates a need for in-depth exploration of alternative methodological approaches and data collection instruments to investigate diarrheal disease in various contexts.

Further exploration of illness representations reported by children and adolescents is also important for assessing WASH studies involving adolescents. Studies have shown that health assessments by adolescents are susceptible to measurement error, because their illness representations may be influenced by accelerated physical and psychosocial developmental changes (27–30). Therefore, perceived health status is enhanced if adolescents perceive data collection tools as acceptable and helpful in self-reporting.

The present study addresses the development of an enhanced understanding of diarrheal illness and its corresponding self-report challenges among this age group. It is also important to note that this study focuses on diarrheal illness rather than diarrheal disease, because illness is a more subjective interpretation of ill-health, instead of objectively, pathologically, or medically defined and measured disease (31–34). Furthermore, illness representations are cognitive beliefs that an individual develops to structure their perceptions about an illness, which often include multidimensional components that identify symptoms, severity, duration, and cause of an illness (35). Here, we apply the Common Sense Illness Representation Model (CSIRM) (35) which is an explanatory model to assist in the exploration of individuals' illness representations (36, 37).

In an effort to improve approaches for measuring self-reported diarrheal illness among adolescents for a school-based WASH health promotion intervention and to exemplify youth-focused intervention adaptation processes, we conducted a small-scale descriptive study as part of the formative research phase in the cultural adaptation of Project SHINE (Sanitation and Hygiene INnovation in Education) (38).

To the best of our knowledge, this is the first study that investigates diarrheal illness representations using the Bristol Stool Form Scale (BSFS), among adolescents. The aims of this study are two-fold. Firstly, to understand cultural representations and perceptions of diarrheal illness among local adolescent students from the rural community of Thirumalaikodi, Tamil Nadu, India. Secondly, to explore whether the BSFS was suitable for local adolescent students from this community (acceptability).

## Materials and Methods

### Study Design

In this study, we employed a convergent parallel mixed-methods study design (39). Creswell and Clark (38) explain that this design enables the researcher to develop a more “complete understanding” of a given phenomenon. Quantitative and qualitative data were collected concurrently, then data underwent separate analysis processes. This study was also based on criteria for studies that explore acceptability articulated by Bowen, Kreuter (40) and as such, there is a greater emphasis on the qualitative component presented in this manuscript.

### Study Setting

The study was conducted from October 2017 to July 2018 at one school (grades Kindergarten- 12) in Thirumalaikodi, a village in the Vellore district in the southern Indian state of Tamil Nadu. The Vellore district has a population of approximately 3.9 million with a majority being Tamil-speaking and Hindu (41). Other predominant languages are English and Telugu. The district is primarily rural and is made up of 858 villages and 36 towns each under the authority of 22 Town Panchayats (41). Additionally, located within the area is spiritual leader, Sri Sakthi Narayani Amma and the Sripuram spiritual park devoted to Sri Lakshmi Narayani informing the community's strong cultural and spiritual values.

### Sampling/Recruitment

All participants were enrolled at the school in which the future Project SHINE intervention took place. Project SHINE is a health promotion intervention that aims to improve sanitation and hygiene-related knowledge, attitudes, and practices among students and teachers through the application of community-based participatory research (CBPR) and participatory science (38). Therefore, adolescent study participants were representative of future SHINE intervention participants. Since Project SHINE is a CBPR intervention, it is important that participant and community input was considered in the adaptation of intervention content and evaluation tools, to ensure cultural appropriateness. Adolescents were selected to participate in this study, as part of the formative work conducted jointly with youth, the target population of the future SHINE intervention, to promote knowledge co-creation, and to increase understanding of local youth perspectives. The school was selected based on interest expressed by the local community, and a partnership between researchers and local institutions (e.g., schools, hospital, and spiritual leadership).

Schools and students included in this study were selected according to the following recruitment strategy: first, the school was selected from the set of schools invited to participate in the larger intervention study. We obtained approval to conduct research activities and implement the intervention from gatekeepers and local leadership. The school principal subsequently assigned a local community liaison (a schoolteacher), to work with the research team and was responsible for coordinating recruitment, collecting diaries from participants, and scheduling the survey and interviews.

Secondly, a purposive sampling strategy was employed to recruit participants for the study. The community liaison was assisted by ninth standard class teachers to invite students to participate in the study who met the following selection criteria: (1) currently enrolled at intervention school; (2) in the ninth standard; (3) comfortable expressing themselves in English; (4) open to sharing experiences about diarrhea and health; and (5) willing to provide assent to participate and obtain written parental/caregiver consent.

Teaching staff at the school informed students about the purpose of the study before inviting students to participate. Efforts were made to ensure similarity between study participants and future intervention participants because findings from this study would inform survey development, which all intervention students would then complete. Therefore, the research team focused recruitment criteria on participants who attended the same school, ensured an equal distribution of sex (although more girls than boys agreed to participate), and geographical background (all from the Vellore district). In total, 15 students agreed to participate. Of the 15 students who agreed to participate, one was excluded from the survey and diary data collection activities since they were absent from school during the time the survey was conducted. Table 1 shows the participants' demographic characteristics.

**Table 1:** Participants' demographic data; adolescents' perception of diarrhea study, Tamil Nadu, India.

**Variables**	**Categories**	**Number of participants**	**Percentage**
Sex	Female	9	64.3%
	Male	5	35.7%
Age	13 years	4	28.6%
	14 years	10	71.4%
Education level	Grade 9	14	100.0%
Water source (at home)	Tap or piped	8	57.1%
	Rainwater	0	0.0%
	Lake or pond	0	0.0%
	Water truck	1	7.1%
	Water vendor	1	7.1%
	Dug well	0	0.0%
	Tube well or borehole	7	50.0%
Diet	Vegetarian	0	0.0%
	Non-vegetarian	14	100.0%

*Three participants reported more than one water source.

Prior to data collection activities, the research team conducted additional research activities with other groups of students at the school. This means that although there would be a level of awareness about the project in general, none of these students had participated in any other project activities. Therefore, in order to build trust between researchers and study participants, the research team utilized assent processes and an introduction meeting (which explained the aims of this research study and provided additional information on confidentiality) to nurture rapport between researchers and participants.

### Bristol Stool Form Scale

Stool form is an essential criterion for the assessment of diarrheal illness (24, 42). Visual and descriptive tools that evaluate stool consistency may help improve and standardize measures and potentially improve self-reported diarrheal illness among adolescents (43). One such visual tool, the BSFS, was developed by Lewis and Heaton (44). The BSFS ranks images of stool on a scale of 1–7 based on consistency from Type 1 (separate hard lumps) to Type 7 (watery). Type 6 and Type 7 are classified as diarrhea (45, 46).

Although the BSFS was originally developed and validated in Bristol, United Kingdom for adults with irritable bowel syndrome, this study aims to explore its acceptability with a different contextual setting, population, and health challenges. As mentioned previously, the application of the BSFS in WASH research could reduce several types of error related to bias (recall and social desirability) and comprehension (standardize the definition of the disease under investigation) (19, 47). Therefore, we set out to explore if the use of the BSFS to self-report bowel movements among adolescents was acceptable from the perspective of prospective intervention participants.

### Data Collection

#### Qualitative Data Collection

Qualitative research methods were applied to obtain a better understanding of adolescents' knowledge, perceptions, and practices relating to diarrheal illness (48). Semi-structured qualitative interviews were conducted with 10 students (seven females and three males). Interviews aimed to gain insights on cultural representations and perceptions of diarrheal illness and the acceptability of the BSFS as an assessment tool for self-reported diarrhea. Acceptability was explored through interview questions regarding attitudes and opinions about experiences filling out stool diaries which were based on the BSFS. Participants were purposively sampled and recruited by the community liaison.

A semi-structured interview guide was prepared based on the CSIRM (35). The CSIRM is an explanatory model that assists in the exploration of “lay views” or individual illness representations (36, 37). Interview questions explored constructs of the CSIRM [identity, cause, timeline, consequence, cure (35) (see Table 2)] and the BSFS to investigate if the use of the instrument was comprehensible, culturally appropriate, and acceptable in this context (see Table 2). Questions included open-ended questions about the terminology used to describe diarrhea, symptoms, perceived causes, and control/cure (50).

**Table 2:** Overview of CSIRM and corresponding interview questions asked [adapted from (49)].

**Construct from CSIRM**	**Example of questions asked**
Identity	What is the difference between diarrhea and other types of poo?
Cause	How do you think someone might get diarrhea?
Timeline	Can you tell me what happens when someone gets diarrhea? What happens next?
Consequence	What kinds of problems might they have?
Control/Cure	What would they need to do to get better?

All qualitative semi-structured interviews were carried in English out by the first author [redacted for peer review] who has previous experience in qualitative research methods. Interviews were conducted at school in a private classroom, lasted from 20–40 min, and were audio-recorded with the consent of participants. As previously mentioned, during interviews, one participant indicated that they were uncomfortable being recorded and the audio recorder was subsequently shut off. Instead, the interviewer [redacted for peer review] took detailed notes for this participant. Data collection continued until 10 participants were interviewed and data saturation was achieved, thus no new information was generated.

#### Quantitative Data Collection

Quantitative data were collected through interviewer-administered face-to-face surveys (*n* = 14) and one-week stool diaries (*n* = 14). The sample size was determined by the study aims which were to explore acceptability, therefore we determined a small sample would be adequate (51). Interviewer-administered surveys were conducted by two female researchers from the local nursing college (SG and RS). Surveys were conducted at the school in a private classroom with blinds/door shut, thus no students were observed during the process. The survey was adapted from a previous BSFS validation study by Guled (52) to appeal to the specified research aims and target population of this study. It included questions concerning demographics, diet, and perceptions of stool using BSFS. Participants were also asked to report on their most recent stool, according to the BSFS. Participants were then requested to complete daily stool diaries for 1-week which used the BSFS to assist in the standardization of illness representation. Furthermore, the application of the BSFS in the diaries and survey triangulated data to broaden our understanding of the self-reported stool form and diarrhea among adolescents in this context.

### Data Analysis

#### Qualitative Analysis

In the qualitative portion of this study, we sought to increase our understanding of diarrheal illness and reporting practices from the perspective of those experiencing it. Data were analyzed using directed content analysis (53), which employs the deductive use of theory to guide analysis. This approach is particularly useful to explore multifaceted and sensitive topics such as ours (54). Here, the CSIRM provided a foundation for the initial codebook and coding scheme (55–57). In order to explore the BSFS acceptability, attitudes regarding the BSFS were also segmented and coded. All interviews were conducted, coded, and analyzed using qualitative data analysis software, Atlas.ti software version 7 (Atlas.ti GmbH, Berlin) by the first author [redacted for peer review]. Transcription was conducted by the first and last author [redacted for peer review]. First, we read text transcripts to get a sense of the whole, then we segregated text into meaning units which were identified and categorized using predetermined categories/themes (informed by the CSIRM and the BSFS). Finally, all text that did not correspond with any of the predetermined categories were given a new code. An example of coding is shown in Table 3.

**Table 3:** An example of the qualitative analysis process.

**Meaning unit**	**Codes**	**Category**
*First of all, we need to [tell] my*… *our, parents, because they are taking care of us. Our parents or grandmas will be using some of the medicines to cure it (Participant 1)*.	Communication with others Culture Family	Cure/control

#### Quantitative Analysis

Our quantitative investigation aimed to summarize and organize salient information on cultural representations and perceptions of diarrheal illness and the BSFS as a tool for self-reporting in WASH interventions involving adolescent participants. We used the SPSS software version 25 to analyze the data (SPSS Inc, Chicago, Illinois). Descriptive statistics were used to summarize survey questions and demographic variables.

### Ethical Considerations

The study protocol was approved by the Norwegian Centre for Data Research (reference number: 2017/53162) in Norway and the Institutional Ethics Committee/Institutional Review Board at the Sri Narayani Hospital and Research Centre (reference number: 30/25/02/17) in India. Informed assent and informed active parental consent were provided for all participants. Furthermore, since this study dealt with a sensitive topic, participants were invited to ask questions, decline to answer or switch off the audio-recorder at any time.

## Results

### Qualitative Data

Interviews generated 29 codes which were then organized into categories according to the constructs of the CSIRM (identity, cause, timeline, consequence, cure) and the BSFS. Quotes have been edited for readability with all changes denoted by square parentheses.

#### Identity

Illness representations generally contain an identity component, which includes the name of the illness and identification of various symptoms that an individual believes are associated with the condition. When discussing diarrhea, participants revealed that the identity dimension included diarrheal illness' physical characteristics, symptoms, and beliefs about the cause of diarrheal illness. Diarrheal illness was defined as “loose motion”, “going in liquid fluid”, or “fecal matter in liquid state.” However, identification of diarrheal illness often included descriptions of related symptoms such as “stomach ache,” “feeling bored,” “can't sit,” “always be in restrooms,” and “fever” and perceived causes such as “bacterial infection,” “vitamin deficiency,” and “consumption of unhealthy foods.” For example, one participant shared:

*Diarrhea is the frequent stools [that] we get when we eat contaminated food, which is not suitable to our health. So [it] doesn't get digested properly (Participant 2, Female)*.

This demonstrates that participant definitions of diarrhea included a combination of several CSIRM constructs (e.g., cause and consequence).

Participants also commented that in parallel with identification, communicating to others about diarrheal illness is difficult. This demonstrates how the *identification* dimension and *consequence* are closely linked. One participant shared that he dislikes talking about diarrhea because of its defining characteristics. He goes onto explain that he perceives diarrhea as, “ugly” and having a “bad smell” (Participant 9, Male).

#### Cause

Although a few participants struggled to describe the cause of diarrhea, others suggested several potential causes such as hygiene, bacterial diseases, and consumption of contaminated food, roadside water, hair, spicy food, or eating non-vegetarian food. One participant shared:

*If they eat junk foods, what they eat [does] not [get] digested [and] vomit or diarrhea will come. [Or] when you're eating some hair. Hair goes into our stomach and causes diarrhea (Participant 4, Female)*.

Here the participant discusses how consumption of certain food types (e.g., junk food) causes diarrhea.

Another participant also talks about how she believes hygiene can also cause diarrhea when she says,

*Someone might get sick when they are not having [good] personal hygiene (Participant 1, Female)*.

Here the participant discusses behavioral causes of diarrheal illness and links personal hygiene.

#### Timeline

It is also important to acknowledge what was difficult for some participants to discuss throughout interviews. Some participants often answered questions in a single word or avoided implicating themselves in discussions of diarrhea (e.g., avoidance of revealing personal experiences). This distanced participants from speaking about the topic in-depth. This was illuminated when participants discussed the timeline dimension since this question invited further reflection on an individual's personal experience with diarrhea illness in terms of frequency and duration of diarrheal episodes. Participants' characterization of a diarrheal illness duration varied, occurring within a 1–5-day period. Some participants also specified that after 2–3 days they should seek help from a healthcare professional. Participants' reports of frequency also varied ranging from 1–3 times a day to 6–7 times a day.

#### Consequence

Illness representation constructs include beliefs about the consequences of the condition for an individual. Here, participants revealed that the consequence dimension was multifaceted and complex. Participants discussed several physical symptoms of diarrhea in addition to other dimensions of everyday life that related to their inability to participate in certain activities, attend school, or communicate with others based on the burden of diarrheal illness.

Participants discussed how they felt uncomfortable talking to others about diarrhea including family and friends. One participant shared:

*I will feel very shy to talk about it to anyone (Participant 10, Female)*.

This shyness is echoed by others:

*Well [when] someone gets diarrhea means they feel they [cannot] talk freely. In that time, they'll have some problems… stomach pains…diarrhea, no? so they cannot talk freely with us. They feel shy to share (Participant 4, Female)*.

Here, participants discussed how they do not feel comfortable talking to others about diarrhea especially with friends at school.

Participants revealed that diarrhea was stigmatizing and influenced participation or performance in school and/or relationships. One participant shared:

*A person with diarrhea will not be normal because they might not know when we get stools. We have the sense it is going to come, but [with] diarrhea we might not know and sometimes the people may not come out of their houses in our places because they will think when we go out, we can't use the proper washrooms. So, they stay inside (Participant 2, Female)*.

In this excerpt, the participant describes how diarrhea threatens normalcy because of the uncertain nature of diarrheal illness. This uncertainty causes one to stay home because they will not know where and when they will be able to use a sanitary toilet.

The same participant continues to discuss how diarrhea affects their sense of normalcy:

*We can't behave as we [did] before. First thing, a person will feel uncomfortable and he just feels if “it” comes out, what will happen so, he can't [go] use. Some people have the habit of using washrooms only in their homes because it will be easy for them. Some people [are] using everywhere, and they get afraid of infections… what they get when they use a public toilet (Participant 2, Female)*.

Here, this participant shares that when a person has diarrhea, they will feel uncomfortable and stay home in order to reduce the risk of using a public toilet which they perceive as dangerous or unhealthy.

This participant then further elaborates about why people are hesitant or afraid of using public toilets.

*In their house they maybe have a personal toilet, but outside, every person, is using a [public] toilet which, they may feel uncomfortable to use. Even I don't use in every place. We get infections in the toilets which are public. Everyone [uses them], so after[wards] they don't keep it [as] clean as it was before. They just complete their “work” (diarrhea) and move. They don't clean up inside. So, in India, it is a very very big problem. They just come finish their “work” [and] go off. So, that is why people think not to come out and use public toilets (Participant 2, Female)*.

Participants also spoke about how diarrhea can disturb their participation at school. One participant reflects:

*In these break times we have the habit of going to washroom, but in the class, if we start disturbing the teacher and going out and coming back, everyone will think, what are they doing? Just going for every single period, everyone will be distracted [by that] particular person, so they'll start asking questions (Participant 2, Female)*.

This account suggests that consequences of diarrhea influence toilet use behaviors that may be deemed suspicious by peers.

Participants often reflected on toilet use behaviors during a diarrheal episode. Here, one participant connects the unpredictability or uncertainty of diarrhea with open defecation.

*[In the villages] I see some of them have toilets and some of them are going in [the] forest, they have a small forest which [is] full of small small trees. They go in the forest and release their diarrhea and come home and washing it. [They go in the forest because] their house is here, and their land of cultivating is far away. When [it's] one kilometer [to the toilet] … [and] they got diarrhea when they [are] cultivating land they can't come to the house, so they go in [that] place (Participant 9, Male)*.

Diarrhea was often characterized as urgent or unpredictable (e.g., “can't sit”). Here this participant connects symptoms to open defecation.

#### Control/Cure

Participants spoke of several diarrheal treatment methods and coping behaviors when discussing the cure dimension of illness representations. These methods can be classified into three categories which included diet modifications, medical and naturopathic treatments, and communication with others about illness (see Table 4).

**Table 4:** An overview of cures reported by participants in semi-structured qualitative interviews (*n* = 10).

**Dietary modification**	**Medical and naturopathic treatments**	**Communication**
• ‘Eat healthy foods such as soft vegetables’ • ‘Avoid [certain] foods [like] butter, oil, and street foods like *pani puri*’ (*Panipuri* is a deep-fried snack that is filled with water that is often sold from roadside food vendors) • ‘Avoid spices like chili powder’ • ‘[Eat] mostly curd’ • ‘Avoid rice’ • ‘Juice milkshakes, fruits, vegetables are good [to eat]’ • ‘Eating [food] raw is good’ • ‘Avoid all the type of sambal foods’ • ‘[Avoid] the gas foods which we eat, the carbonated things, food that has spices like *dhal*’ (*Dhal* is a traditional soup made from lentils) • ‘Give fruit juices curd rice and *chapatti*’ (*Chapati* is a traditional flatbread dish that is commonly eaten in India) • ‘[Eat] fruits like apples’ • ‘Hot water we should drink’ • ‘Avoid junk foods in night times’ • ‘Eat cold items’ • ‘Sugar and water is the easiest method to cure the diarrhea’	• ‘Taking a tablet after their meal’ • ‘Go to the hospital’ • ‘*Siddha*’ (*Siddha* is a traditional medicine system which has its origins in Tamil Nadu) • ‘*Ayurveda*’ (*Ayurveda* is traditional medicine that can be traced back to the Vedic period) • ‘Medicinal plants’ • ‘Serum *cumin pani*’ (*Cumin pani* is a traditional remedy used for digestion) • ‘Syrup from the hospital’	• Parents • Doctors • Teachers

For diet, participants shared that it was important to “eat healthy foods such as soft vegetables” (Participant 1, Female) and “avoid [certain] foods [like] butter, oil, and street foods like *pani puri*” (Participant 2, Female). Several participants also discussed how spicy foods were the cause of diarrhea therefore many suggested that one must, “avoid spices like chili powder” (Participant 2, Female) to alleviate symptoms. Medical treatments included both modern and traditional remedies such as, “the hospital” (Participant 5, Male) and “Ayurveda” (Participant 1, Female). Furthermore, participants also discussed how communication with doctors, teachers, or parents was an essential step in treating diarrheal illness.

Although participants discussed difficulties in speaking about diarrhea, participants shared that they felt open to communicate with their parents about diarrheal illness. One participant shared:

*Parents will get the person to the hospitals and medicines will be given and they take care very carefully. If there are any problems the parents will uh cure that (Participant 3, Male)*.

Another participant explained:

*First of all, we need to [tell] my… our, parents because they are taking care of us. Our parents or grandmas will be using some of the medicines to cure it (Participant 1, Female)*.

This was further elaborated upon, describing how parents are able to help cure diarrhea.

*We must say to our parents so that they can stop [it], they can give tablets, or give an aid to our doctor. Saying to our parents is important, but not to our friends (Participant 2, Female)*.

Several participants discussed how they preferred to talk to their mothers specifically about diarrheal illness. This participant explained:

*Firstly, she is also a woman and that's the point and the second thing is that I share everything to my mother. So, it includes my personal things and personal hygiene. So, I say to my mother first, and then she gives a measure to it (Participant 2, Female)*.

Some also discuss willingness/openness to communicate with doctors about diarrhea whom they perceive as the gateway to effective treatment.

*Once we say everything to a doctor, he can judge what's the problem. So, like we say the color and what state it is coming out and when how many times. So, once we say everything to a doctor in a correct manner he can give and suggest a correct medicine or something to us. So that is why it is easy to say something to a doctor (Participant 2, Female)*.

One participant discussed how as he gets older it becomes more difficult to talk about illness with his mother. While another participant echoes similar sentiments:

*If I speak about it, someone will be laughing. So … I don't like to talk of that. I will feel ashamed to say to my mother also because I am 14 years. Up to 10 years I will say to my mother without [feeling] shy, [but] now I'm grown up so I feel ashamed to say like this (Participant 8, Female)*.

Unlike others, this participant talks about how fear of teasing prevents her from sharing when she has diarrhea with her mother.

#### Bristol Stool Form Scale

Another category relating to the study's aims pertaining to the BSFS was extracted from interviews with adolescent participants. Although participants unanimously reported that the BSFS was easy to understand, their experience using the BSFS in the stool diaries was mixed. Some participants discussed how they felt uncomfortable reporting their stool form using the form scale. For example, one participant shared:

*Because it is a personal thing about us, and we can't say it freely of course. So, it seems somewhat bad for me*…*I just feel somewhat bad to fill it, but it is our health issues, so I feel good too. Sometimes it will be so shy for me, and otherwise, it's not, other things it's OK* (Participant 1, Female).

In another account, one participant reflected:

*I feel shy to fill out that form. [When] mam came [with] that form, everyone [was] asking what is in that envelope. We all felt ashamed to say what [it was] because it is [about] stool. If you say that it is a stool form [that] we have to write [and] we have to mention what type is it, if we say that to our friends, they would be laughing. So, we felt ashamed to say that*. (Participant 8, Female).

While some participants shared, they felt shy or embarrassed to fill out the stool diaries which applied the BSFS, others reflected on the potential benefits of recording their health information:

*First, I thought, why should I do this? First thought it's something person[al] to us, so why should I give it to someone else? And later on, I thought it's somewhat helpful for everyone because sanitation and hygiene is so important for girls especially. So, giving out this makes something useful like this. So, first time it was something awkward to give, the next day and the next day after that I thought it was good, so I [did] it properly* (Participant 2, Female).

Furthermore, another participant continues to evaluate the potential value of the diary when sharing the following excerpt.

*What's OK is we can keep a good schedule for that, when we are eating something that's bad it will be, leads to some bad response of poo. When we are somewhat eating good foods it seems normal, normal motion, so that's the good thing* (Participant 1, Female).

As described previously in Table 4, participants described foods to avoid which included spicy foods, butter, oil, and other street foods. Contrastingly, good foods were classified as healthy, fresh, and often associated with vegetables. The excerpt presented here also highlights the potential benefits of the implementation of a stool diary.

### Quantitative Data

Participants had a mean age of 13.7 years (range 13–14) and over 60% of participants were female (Table 1) and all reported that they eat meat (non-veg). Tap or piped water was the most commonly used water source (57%) then tube well or borehole (50%), followed by water truck and water vendor (both 7%).

We asked participants to self-report their stool form/bowel movements (frequency and consistency) using the BSFS (see Tables 5, 6). Every participant (100%) answered questions requiring them to self-report stool form. Furthermore, all participants reported that they were able to match different types of stool to the pictures on the chart. Students were then asked to use pictures to describe their most recent stool, 64% selected Type 3, 21% selected Type 4, and 14% selected Type 6. Participants also used the BSFS to assess healthy stool for different populations (Table 6). Ten out of 15 or 67% of participant responses ranked Type 4 as healthy stool for someone of their age (13, 14) and 73% of participant responses ranked Type 7 as an unhealthy stool.

**Table 5:** Overview of variables in the survey.

**Variables**	**Categories**	**Number of answers**	**Percentage**
Last bowel movement	Today	14	100.0%
	Yesterday	0	0.0%
	Other	0	0.0%
Bowel movement frequency (yesterday)	1 time	9	64.3%
	2 times	3	21.4%
	3 times	1	7.1%
	0 times	1	7.1%
Bowel movement frequency (day before yesterday)	1 time	11	78.6%
	2 times	1	7.1%
	4 times	1	7.1%
	5 times	1	7.1%
Bowel movements visible	Yes	8	57.1%
	No	6	42.9%
Last bowel movement seen	Today	8	57.1%
	Yesterday	3	21.4%
	Day before yesterday	1	7.1%
	No idea/no habit of looking	2	14.3%
Ability to match stool on BSFS	Yes	14	100.0%
	No		
Which type of stool according to BSFS resembles most recent bowel movement	Type 3	9	64.3%
	Type 4	3	21.4%
	Type 6	2	14.3%
Cure dimension	Diet	3	21.4%
	Medical/healthcare	10	71.4%
	Home remedies	7	50.0%

**Table 6:** Overview of survey results: participant perceptions of stool using the BSFS.

**Stool type**	**Perceived healthy stool for an adolescent**	**Perceived healthy stool for an infant**	**Perceived healthy stool for an adult**	**Perceived unhealthy stool (general)**
Type 1	0 (0.0%)	0 (0.0%)	0 (0.0%)	2 (13.3%)
Type 2	1 (6.7%)	3 (20.0%)	3 (18.8%)	0 (0.0%)
Type 3	3 (20.0%)	0 (0.0%)	8 (50.0%)	0 (0.0%)
Type 4	10 (66.7%)	1 (6.7%)	1 (6.3%)	1 (6.7%)
Type 5	1 (6.7%)	4 (26.7%)	3 (18.8%)	0 (0.0%)
Type 6	0 (0.0%)	5 (33.3%)	1 (6.3%)	1 (6.7%)
Type 7	0 (0.0%)	2 (13.3%)	0 (0.0%)	11 (73.3%)

Participants discussed various cures for diarrhea (Type 6 and 7) which were then classified into three variables (e.g., diet, medical/healthcare, and home remedies) (see Table 5). Ten out of 14 students (71%) suggested that medical/healthcare strategies were a good cure (e.g., taking tablets, visiting the doctor, or hospital), 50% recommended home remedies, while 21% of participants indicated that changes in dietary intake would help cure diarrhea.

Stool diaries gave insight into the participant's everyday stool. All participants who completed the survey rated stool for 1 week on the BSFS's seven-point scale and recorded how many times they had a bowel movement. Results are shown in Table 7 and display the first recorded values. In total, participants recorded 111 stools over a seven-day period (participants averaged 7.92 bowel movements per week (ranging from 7 to 10) or 1.1 stools per day. The most common types of stool rated in surveys (last bowel movement) was Type 3 at 63%, while diaries were Type 4 at 38%. Furthermore, 14% of study participants reported their last bowel movement as diarrhea (Type 6 or 7) in the survey while 10% of total stools reported in the diaries could be classified as diarrhea. In addition, eleven participants reported that they had diarrhea at least once over the 1-week period, however, no participants reported diarrhea (Type 6 or 7) more than 3 times in a 24-h period which would align with the WHO's classification of diarrhea.

**Table 7:** Overview of total number of self-reported stool type in diaries.

**Stool Type**	**Number**	**Percent**
Type 1	3	2.7%
Type 2	6	5.4%
Type 3	34	30.6%
Type 4	43	38.7%
Type 5	14	12.6%
Type 6	11	9.9%
Type 7	0	0.0%

## Discussion

This study explored cultural representations and perceptions of diarrheal illness among adolescents and the acceptability of the BSFS. We found that cultural representations and perceptions of diarrheal illness and individual health assessments that influence self-report among adolescents are linked to attitudes and beliefs that are both individual and context-specific (e.g., individual beliefs about the consequences or cause of diarrheal illness and traditional diarrheal treatment methods). Other research has also contributed to the cultural understanding of health-related perceptions and representations maintained by local populations in low- and middle-income (58). However, understanding health and illness is vastly complex, therefore it is important to use exploratory research to uncover local perceptions among subsets of populations such as adolescents (36, 37).

In the quantitative portion of the study, all participants demonstrated that they were able to understand the BSFS and apply the scale to their own bowel movements, however, results from the qualitative component reveal that taboos may influence how diarrheal illness is reported to outsiders. Participants' perception of healthy and unhealthy stool was consistent with previous research. One study previously reported that the average BSFS score for healthy adults was 3.6 (59), while the average BSFS score for the present study was 3.8. Results from the surveys showed that the most commonly reported stool type was Type 3 (63%), while the corresponding number for diaries was Type 4 (38%). This is also consistent with another study in east India which reported that the most predominant stool form reported by study participants was Type 4 (60) and a study from the southern Indian city of Chennai which reported that the most common stool types were Type 3 and 4 (61). Furthermore, literature has demonstrated that stool frequency between 3 per week and 3 per day is normal (62). Therefore, the participants' reported range of frequency of stools per week was within an acceptable range also when compared to other research on stool frequency and form in India (60).

According to the survey results, diaries, and interviews, diarrhea (Type 6 and 7) is an irregular occurrence. Therefore, it can be interpreted that adolescents who participated in this study, do not perceive diarrhea as normal or healthy. Moreover, 73% of study participants indicated that they perceive Type 7 (watery stool) as illness-related. This is closely related to the *identity* dimension of the CSIRM which pertains to labeling the illness and knowledge of illness symptoms (37). In semi-structured qualitative interviews, participants most commonly identified diarrhea by its physical attributes (“loose motions”), however, since physical symptoms are also perceived as one possible component of an illness (28), participants often described diarrhea in symptom-related terms (e.g., fever, stomachache, and fatigue).

The *cause* dimension is connected to beliefs related to factors causing illness or disease (37). These can be biological, emotional, environmental, or psychological (62). In the survey and semi-structured interviews, participants struggled to identify causes of diarrhea. In some interviews, participants discussed various causes of diarrhea, which were connected to hygiene, diet, and bacterial diseases. In a study regarding beliefs about diarrhea causes in another village in Vellore, 43% of participants believed food caused diarrhea and 15.5% did not know the cause (63). Insufficient understanding of the actual causes of diarrhea may foster shame and embarrassment surrounding the illness. Therefore, there needs to be further exploration into discourses about diarrhea to dismantle taboos and inform a culture of accurate and unbiased reporting of diarrheal illness.

The *control/cure* dimension describes how coping behaviors and efficacy of treatment can cure an illness (37). In both surveys and semi-structured interviews, students reported similar cures such as diet, medical intervention, and traditional naturopathic treatments or “home” remedies. Studies exploring cultural representations and perceptions of illness, often include local beliefs and cultural knowledge about illnesses, including descriptions of traditional practices that help treat or cure diarrheal illness (34, 58, 64). Consistent with the literature on traditional medicine in India, cures and/or treatment strategies mentioned by participants can be linked to several ayurvedic practices that are prevalent in the area. Since ancient times, traditional beliefs have widely influenced treatments of various ailments throughout India, therefore, India has a rich tradition in naturopathic medicine systems such as Ayurveda, Siddha, and Homeopathy. Furthermore, findings derived from this study reveal that this local context is no exception. For example, according to Ayurvedic medicine, diarrhea could be caused by a weakened digestive fire (often caused by excess *pitta*^1^) (65). In this study, participants discussed several traditional remedies to help treat diarrhea, that when compared to resources on Ayurveda, also center around medicinal plants and other energetically “cooling” foods, such as fruits, vegetables, and “cold items” (all *pitta*-countering) (65). Moreover, although not all students discussed Ayurvedic medicinal treatments explicitly, several of the cures suggested by participants (such as avoiding the consumption of hot, spicy, or oily foods) are perceived to further weaken digestive fire, which may aggravate the *pitta* (65). However, in the survey, 71% of participants suggested that an individual should seek medical care or pursue other healthcare-related strategies. However, what was noticeably absent in survey results was the mention of communication as a *control* strategy. In the semi-structured interviews, communication (with parents) was often discussed as an initial control strategy or coping behavior. However, communication pathways were inconsistent between participants. While many emphasized the importance of telling their parents when they were suffering from diarrheal illness, some shared that they were uncomfortable speaking to their mother about diarrhea as they grow older. One participant even implied that her mother teased her if she got ill, which led to the avoidance of sharing her health status. However, many participants shared in their interviews that it was vital to share with their parents when they got ill because they described their parents as their caretaker and well-positioned to be able to assist in the *control/cure* dimension of diarrheal illness based on knowledge and previous experience.

The *consequence* dimension of the CSIRM represents beliefs related to the overall impact on an individual's quality of life or functional capacity (37). For example, many assess this dimension according to their inability to perform certain tasks based on illness (37). Findings from semi-structured interviews reveal that participants perceived diarrhea as problematic because it compromises their appearance and participation in everyday activities. These prohibiting qualities that conflict with participant's perceived “normal” every day, included not being able to communicate with others “freely”. Participants expressed a general discomfort with the subject matter and often attributed their apprehensiveness to speak about diarrheal illness to shyness. One participant explained that diarrhea was disgusting and therefore was not something he liked to discuss or share. However, several students shared that they were open to speaking to select individuals about diarrhea such as parents or doctors. Given these contrasts, it appears the premise of communication regarding diarrheal illness is both influenced by taboos and transactions (e.g., I will tell you if you can help me get healthy again). Since peers often are unable to assist with the *control/cure* dimension or treatment, participants appeared hesitant to share. Instead, participants shared that peers may tease them for having diarrheal illness. This is consistent with other research on toilet use which ascertains that bullying is associated with toilet use behaviors at school (66). Moreover, when using the BSFS it is important that researchers clearly explain to participants how accurately reporting diarrheal illness can be mutually beneficial to enhance future WASH research validity.

Willingness to communicate with others may have affected participant's engagement with the BSFS, which varied. Some participants expressed that they were embarrassed to report their stool form/bowel movements in the stool diaries, while others noted the utility of the exercise, using it as an opportunity to understand more about their own health. Moreover, participants indicated that although the BSFS was easily understood, diarrhea itself was uncomfortable to speak about. Anthropological literature that focuses on illness perceptions suggests that cultural representations and perceptions of illness are influenced by the local cultural, contextual, and social environment (31–34, 67). Therefore, it is important to reflect on participants' comfort level with the content of the material (e.g., images and subject matter), especially when relying on self-reported findings to determine intervention effectiveness.

Furthermore, in semi-structured interviews, participants discussed how toilet habits during diarrhea may influence or threaten academic participation at school. Students related frequent toilet use to diarrheal illness, explaining that as an unpredictable illness, a student may experience limited control over where and when they can go to the bathroom. Moreover, participants reported that instead of using a toilet that they deemed unclean or unhealthy, they elected to stay home during bouts of diarrhea to minimize the perceived risks (using a dirty toilet/teasing from peers). Here, the necessity of using an unsanitary toilet facility may further stigmatize diarrhea because, in India, toilets are seen as dirty or impure (68, 69). Other studies have indicated that public toilets are often dirty or poorly maintained (70, 71). In addition, one study also indicated that the smell of public toilets contributed to the preference for defecating in the open (63).

Participants also discussed how they were especially uncomfortable having diarrhea at school because it might be revealed to others that they have diarrhea. Findings reveal that participants were embarrassed to reveal diarrheal illness to others (especially at school) because they perceive diarrhea as disgusting, foul-smelling, unclean, or bad (e.g., physical stigma). Given these aversions, students discussed how diarrheal illness may be perceived as detrimental, prompting teasing from peers, teachers, and/or parents. Participants also shared how they worried that diarrhea at school could cause bullying, therefore they kept it a secret or stayed home. This highlights how secrecy surrounding diarrhea promotes protection from bullying and shame.

We also observed how adolescents perceived, interpreted, and reported diarrhea because of various emotional and cognitive differences (28, 72). Participants discussed how diarrhea can lead to absenteeism or withdrawal from social activities. Previous studies on adolescent illness representations suggest that definitions of illness are often compounded by one's lack of ability to do required and desired activities, such as attending school, sports, and engaging with peers (28, 35, 73). This is influenced by cognitive and developmental changes that influence adolescent's conceptualization of illness and indicators of good health. Because adolescents are currently going through other developmental changes, they may lack cognitive understanding of a disease, that an adult may have (28).

### Strengths and Limitations

There are several strengths and limitations to the study. One strength is the mixed-methods approach that was applied to gain an in-depth understanding of cultural representations and perceptions of diarrheal illness among adolescents. However, the convergent parallel design prohibited insights gained from one method to influence the other, which is a common characteristic in sequential designs (74).

Limitations include the small purposive sample, rather than a randomized population-based sample that included different states in India. As a result, we are not able to generalize the results to other adolescent populations within the Vellore district or outside. In this study, representativeness was not prioritized, although we tried to recruit a sample that would mirror future intervention participants. Furthermore, the small sample size also prevented us from conducting more complex quantitative analyses. However, since the study was a small-scale descriptive formative research study and more qualitatively weighted, we felt this was an appropriate sampling strategy and sample size.

Language was another limitation. All data collection activities were conducted in English. While English is the language of instruction at the school in which the study was conducted, it is not the native language of study participants. Therefore, words that complement pictures on the BSFS such as “sausage” were not widely understood (this could also be because sausage is not part of the local diet in rural India). Although collecting data in English simplified data collection activities for the research team, language may have caused a loss of meaning in the analysis and communication complications (e.g., short verbal responses) in the qualitative component. Therefore, we recommend the future translation of the BSFS to local languages to enhance comprehension by participants and that future data collection activities are conducted in the native language of participants. Nevertheless, we tried to mitigate these limitations through the incorporation of prolonged engagement and inclusion of local research partners in data collection activities.

## Conclusion

The findings of this study provide important descriptive insights on adolescent illness representations in relation to diarrheal illness, in addition to reporting practices for the SHINE intervention and other WASH health promotion intervention research and literature. Findings also suggest that challenges associated with self-reported data on diarrheal illness may be connected to a general reluctance to discuss this issue among this population, thus likely influenced by taboo and stigma. This is a challenge that is rarely discussed explicitly in WASH health promotion literature, indicating that more research is needed.

## Data Availability Statement

The raw data supporting the conclusions of this article will be made available on request to the corresponding author.

## Ethics Statement

The studies involving human participants were reviewed and approved by Norwegian Centre for Data Research (reference number: 2017/53162) Institutional Ethics Committee/Institutional Review Board at the Sri Narayani Hospital and Research Centre (reference number: 30/25/02/17). Written informed consent to participate in this study was provided by the participants' legal guardian/next of kin.

## Author Contributions

AG-W contributed to research project development and research design, data collection, data management, data analysis, and writing and editing processes of the manuscript. GA contributed project development, research design, data analysis, and editing processes of the manuscript. SG and RS contributed to research project development, data collection, and provided input in cultural components of study design. SB contributed to research project development, research design, and editing processes of the manuscript. All authors have read and approved the final manuscript.

## Conflict of Interest

The authors declare that the research was conducted in the absence of any commercial or financial relationships that could be construed as a potential conflict of interest.

## Footnotes

^1^According to *Ayurveda, pitta* is one of the three life forces (*doshas*) and known to regulates metabolic processes.
