# Corrigendum: Understanding Adolescents' Perceptions of Diarrhea: A Formative Research Study of a Visual Scale to Measure Self-Reported Diarrhea in Low-Resource Settings

**DOI:** 10.3389/fpubh.2021.780587

**Published:** 2021-10-08

**Authors:** Anise Gold-Watts, Geir Aamodt, Subramanian Gandhimathi, Rajamani Sudha, Sheri Bastien

**Affiliations:** ^1^Department of Public Health, Faculty of Landscape and Society, Norwegian University of Life Sciences, Ås, Norway; ^2^Department of Community Health, Sri Narayani College and School of Nursing, Vellore, India; ^3^Department of Obstetrics and Gynecology Nursing, Sri Narayani College and School of Nursing, Vellore, India; ^4^Department of Community Health Sciences, Cumming School of Medicine, University of Calgary, Calgary, AB, Canada

**Keywords:** adolescent, diarrheal illness, Bristol Stool Form Scale, illness representations, water sanitation and hygiene

**Text Correction**

In the original article, there was an error. The names are listed incorrectly in the acknowledgments section. It currently reads, ‘*We would like to acknowledge the contributions and support of teachers, staff, and students who participated and contributed to this research project. In addition, we would like to thank the staff at the Sri Narayani Hospital and Research Centre, [redacted for peer review], and [redacted for peer review]*.’

A correction has been made to Acknowledgments, paragraph 1:

*We would like to acknowledge the contributions and support of teachers, staff, and students who participated and contributed to this research project. In addition, we would like to thank the staff at the Sri Narayani Hospital and Research Centre, Dr. Balaji, Mr. Suresh Babu, Mr. Ramesh Shanmugasundaram, and Mrs. Aruna Ganesan*.

The authors apologize for this error and state that this does not change the scientific conclusions of the article in any way. The original article has been updated.

## Publisher's Note

All claims expressed in this article are solely those of the authors and do not necessarily represent those of their affiliated organizations, or those of the publisher, the editors and the reviewers. Any product that may be evaluated in this article, or claim that may be made by its manufacturer, is not guaranteed or endorsed by the publisher.

